# Phenotypic Profiling of Biofilm Formation and Antibiotic Susceptibility in Poultry-Derived *Listeria monocytogenes* Isolates

**DOI:** 10.3390/antibiotics15060577

**Published:** 2026-06-05

**Authors:** Evangelia A. Karamani, Eirini Kerousi, Margarita Adosidi, Georgios Vafeiadis, Ioannis S. Boziaris, Efstathios Giaouris, Foteini F. Parlapani

**Affiliations:** 1 Laboratory of Marketing and Technology of Aquatic Products and Foods, Department of Ichthyology and Aquatic Environment, School of Agricultural Sciences, University of Thessaly, 38446 Volos, Greece; ekaramani@uth.gr (E.A.K.); boziaris@uth.gr (I.S.B.); fwparlap@uth.gr (F.F.P.); 2 Laboratory of Food Microbiology and Hygiene, Department of Food Science and Nutrition, School of the Environment, University of the Aegean, 81400 Myrina, Lemnos, Greece; kerousieirini@gmail.com (E.K.); margaritadosidi@gmail.com (M.A.); georgevafe@gmail.com (G.V.)

**Keywords:** *Listeria monocytogenes*, biofilm formation, microtiter plate assay, antibiotic susceptibility, food safety, poultry

## Abstract

**Background/Objectives**: *Listeria monocytogenes* is a critical foodborne pathogen, with poultry products serving as a potential reservoir. Its ability to form biofilms may aid in its persistence on processing equipment and food-contact surfaces, while antibiotic resistance complicates efforts to control and treat infections. This study aimed to characterize, in parallel, the biofilm-forming capacity and antibiotic susceptibility of a large collection of poultry-derived *L. monocytogenes* isolates (n = 93) to better understand their potential for persistence and to clarify how the biofilm phenotype may relate to the bacterial antibiotic response and to inform risk assessment and targeted control strategies along poultry processing and supply chains. **Methods**: Biofilms were evaluated on polystyrene microtiter plates at 12 and 30 °C in a nutrient-rich laboratory medium. Susceptibility to eight clinically and food-relevant antibiotics was tested using disk diffusion and interpreted according to European Committee on Antimicrobial Susceptibility Testing (EUCAST) breakpoints when available. **Results**: At 30 °C for 48 h, 69.9% of isolates were classified as weak biofilm formers and 30.1% as non-biofilm formers, whereas at 12 °C for 120 h, 55.9% were weak, 16.1% moderate, and 28.0% non-biofilm formers, with no strong biofilm producers identified under either condition. Overall, the isolates remained largely susceptible to ampicillin, penicillin G, vancomycin, tetracycline, and chloramphenicol, with 87.3% of inhibition zones across all drugs falling within the 20–29 mm and 30–39 mm categories, while small subpopulations showed reduced susceptibility or resistance to trimethoprim–sulphamethoxazole (TMP-SMX) and, particularly, erythromycin and streptomycin. No consistent correlation was found between biofilm-forming ability and antibiotic susceptibility, indicating that these phenotypic traits are largely independent in this collection. **Conclusions**: These findings reveal that poultry-derived *L. monocytogenes* isolates can form weak to moderate biofilms under the tested monoculture conditions while generally maintaining susceptibility to first-line antibiotics. However, the development of macrolide- and aminoglycoside-resistant subpopulations, along with the potential for increased colonization within complex multispecies biofilms in real processing environments, emphasizes the importance of ongoing integrated surveillance across animal food systems.

## 1. Introduction

*Listeria monocytogenes* is an opportunistic, facultative intracellular, foodborne bacterial pathogen that causes invasive listeriosis in at-risk populations [1]. This serious illness has high hospitalization and case-fatality rates, especially among fetuses, newborns, older adults, and immunocompromised individuals [2]. According to the latest epidemiological data for Europe, in 2024, listeriosis was the fourth most reported foodborne illness (zoonosis); however, it was the most severe, with the highest percentages of hospitalizations and case fatality rates (72.1% and 8.3% in outbreak cases, 97.3% and 15.6% in sporadic cases) [3]. These rates are not provisional and have remained high throughout the past years. Its psychrotrophic nature—meaning that it can grow at refrigeration and other low temperatures, as well as at mesophilic temperatures around 30–37 °C—together with its ability to withstand and grow under a wide range of environmental conditions (including high salt concentrations and low pH), and to colonize and persist in food-processing environments make *L. monocytogenes* a major concern throughout the food chain [4].

A key survival strategy of *L. monocytogenes* in food processing settings is believed to be biofilm formation, the development of structured communities of cells embedded in a self-produced extracellular matrix attached to abiotic or biotic surfaces [5]. Cells within biofilms typically display greater tolerance or resistance than planktonic cells to environmental stresses such as desiccation and nutrient limitation, as well as to sanitizers and antibiotics, making eradication in food plants more difficult [6]. Numerous studies have demonstrated that *L. monocytogenes* can form biofilms on common food contact surfaces, including stainless steel, glass, and plastics such as polystyrene (PS), and that biofilm-forming capacity can vary markedly among strains [7,8,9]. Environmental factors—including temperature, nutrient availability, and surface properties—are critical in shaping the extent and architecture of *L. monocytogenes* biofilms [5,10,11].

Alongside concerns about persistence on equipment and product-contact surfaces, the emergence of antimicrobial resistance (AMR) in *L. monocytogenes* isolates from foods and food processing environments has raised additional public health concerns [12,13]. Although *L. monocytogenes* is generally regarded as susceptible to first-line treatments such as penicillin, ampicillin, and gentamicin [14,15], several surveys have reported reduced susceptibility or resistance to clinically and veterinary-relevant antibiotics, including TMP-SMX, tetracyclines, and macrolides, among foodborne isolates [12,16,17]. The co-occurrence of virulence factors, biofilm-forming capacity, and antibiotic-resistant phenotypes in foodborne *L. monocytogenes* strains underscores the risks of cross-contamination in processing environments and hard-to-treat invasive infections [18].

Foods of animal origin are recognized as important vehicles for *L. monocytogenes*, underscoring the need to better understand the traits that promote its survival and dissemination in this sector [17,19,20]. Surveillance data repeatedly show that *L. monocytogenes* can be detected in raw and ready-to-eat (RTE) meat and poultry products, as well as on associated processing surfaces, and that a subset of strains may persist for extended periods despite routine cleaning and disinfection [21,22]. Interestingly, persistent strains in food processing environments have been reported to differ from sporadic strains in traits linked to environmental fitness, such as biofilm formation and sanitizing tolerance, potentially contributing disproportionately to recurring contamination events [23,24]. However, relatively few studies have systematically characterized biofilm-forming ability and susceptibility to clinically important antibiotics in parallel across diverse collections of foodborne *L. monocytogenes* isolates, especially those predominantly from poultry meat and related products [17,25,26,27].

Against this background, the present study examined the biofilm-forming ability of a large panel of *L. monocytogenes* isolates from poultry meat (n = 93) on PS microtiter plates at two temperatures representative of chilled and severe-abuse conditions in food-processing environments (12 and 30 °C), using a crystal-violet assay to quantify biofilm biomass. In parallel, the same isolates were characterized for susceptibility to eight clinically and food-safety–relevant antibiotics by disk diffusion on Mueller–Hinton agar, and the resulting inhibition zones were interpreted according to EUCAST clinical breakpoints when available, enabling assessment of potential associations between biofilm-forming capacity and antibiotic susceptibility profiles. By integrating quantitative profiling of biofilm formation with antibiotic susceptibility testing across a large set of poultry-derived *L. monocytogenes* isolates, this study aims to clarify how the persistence-related biofilm phenotype may relate to the bacterial antibiotic response and to inform risk assessment and targeted control strategies along poultry processing and supply chains.

## 2. Results and Discussion

### 2.1. Biofilm-Forming Potential of Isolates

**Figure 1:**
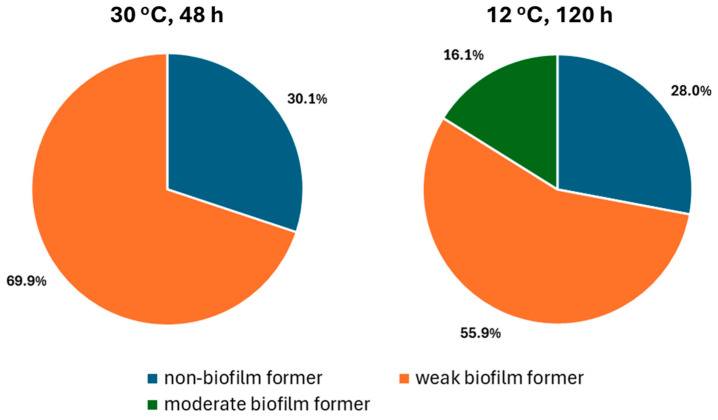
Pie charts showing the rates of biofilm-forming ability (i.e., non-biofilm former, weak, moderate biofilm-former) among the *L. monocytogenes* isolates (n = 93) under the two incubation conditions examined (either at 30 °C for 48 h or at 12 °C for 120 h).

Table S1 provides detailed data on the biofilm biomasses (A_590 nm_) of the 93 *L. monocytogenes* isolates and classifies them as non-, weak-, or moderate-biofilm formers on PS microtiter plates in BHI broth at 30 °C for 48 h or at 12 °C for 120 h. It should be noted that the two incubation regimes used here were selected to reflect realistic processing scenarios rather than to independently dissect the effects of temperature and time; therefore, the biofilm data should be interpreted as condition-specific phenotypic profiles of the tested isolates. As summarized in Figure 1, under both incubation conditions, most isolates produced detectable biofilm but were predominantly classified as weak biofilm formers. Specifically, at 30 °C, 69.9% of isolates were weak biofilm formers, whereas the remaining 30.1% were non-biofilm formers. At 12 °C, the proportions of weak biofilm formers and non-biofilm formers decreased to 55.9% and 28.0%, respectively, while 16.1% of isolates were reclassified as moderate biofilm formers. No isolate met the criteria for classification as a strong biofilm former at either temperature. These results suggest that although many of the tested isolates can colonize and accumulate biomass on PS, they typically form only weak-to-moderate biofilms, and that incubation temperature and time strongly influence biofilm formation. Moreover, Spearman’s rank-order correlation analysis showed that biofilm biomass values obtained at 30 °C and 12 °C were strongly and positively correlated (Spearman’s rho = 0.794, *p* < 0.001), indicating that isolates that form more biofilm under one condition also tend to do so under the other.

A more detailed examination of the category shifts between the two incubation temperatures shows that 15 isolates moved from the non- and weak biofilm-forming groups at 30 °C into the moderate group at 12 °C, consistent with the psychrotrophic character of *L. monocytogenes* and its ability to grow and gradually accumulate biomass at low temperatures [28]. This behavior aligns with previous studies reporting that *L. monocytogenes* can build up sessile biomass on abiotic surfaces at low temperatures (10–12 °C) when nutrients are abundant [5,10,29]. Similar observations have been made at standard refrigeration temperatures (4 °C), further confirming that this pathogen can colonize surfaces under chilled conditions typical of food processing and storage [7,30].

The absence of strong biofilm formers in our panel indicates that these poultry-derived isolates generally exhibit only a moderate intrinsic capacity for biofilm formation in the microtiter plate assay. At the same time, biofilm formation in *L. monocytogenes* is both strain-dependent and highly sensitive to environmental and assay conditions—including temperature, nutrient availability, surface properties, and interactions with other microorganisms—so it remains possible that some of these poultry isolates would display a strong biofilm phenotype under different, more complex conditions typical of processing environments. Nevertheless, this pattern of weak biofilm-forming capacity aligns with earlier surveys of food and processing-environment isolates, in which weak or moderate biofilm formation is predominant, and strong biofilm producers constitute only a minority of strains [31]. For example, in one study of 98 *L. monocytogenes* isolates from diverse sources, most strains were classified as weak or non-formers, with only a small fraction (9.2%) showing strong biofilm formation in a similar microtiter plate system [32]. In addition, previous studies have shown that the biofilm structure, density, and total biomass of *L. monocytogenes* are strongly influenced by nutrient quality and concentration [33,34]. Interestingly, in a study using diluted BHI with a microfluidic or static system, nutrient restriction markedly altered biofilm structure, promoting transitions from compact multilayers to more open, filamentous networks and reducing total biomass compared with full-strength BHI [35]. These findings underscore that the biofilm-forming potential of a given strain in vitro depends not only on its intrinsic capacity but also on the nutritional context during surface colonization.

It is important to emphasize that the limited biofilm formation observed here in vitro, using a standardized microtiter plate assay, does not necessarily mean that a weak or moderate biofilm-forming strain cannot persist in the environment. This is especially true when strains encounter more complex substrates, surface topographies, and multispecies communities than those simulated here [36]. Therefore, although many *L. monocytogenes* strains from meat, poultry, fish, and processing environments have shown only limited biofilm formation in vitro, these strains have still been linked to persistence in facilities [37,38]. On the other hand, in a previous study analyzing 124 persistent *L. monocytogenes* strains repeatedly isolated over 4 years in a meat processing facility in Switzerland, all strains exhibited biofilm formation comparable to that of a high-biofilm-forming strain [39]. At the same time, other studies in food plants have shown that some so-called persistent strains can initiate and develop biofilms more quickly and more intensely than sporadic strains, thereby disproportionately contributing to long-term colonization of equipment and hard-to-clean niches [22,24]. Undoubtedly, from a broader food-industry perspective, even weak or moderate biofilms can enhance stress tolerance and support survival during cleaning and disinfection [40]. Additionally, in real processing environments, pathogenic bacteria are likely to coexist with conditioning films from animal tissues and organic debris, as well as with the resident microbiota, which can either hinder or promote listerial attachment and growth [41]. Taken together, these in vitro findings may help explain the persistence of certain strains observed in meat processing facilities.

### 2.2. Antibiotic Susceptibility Profiles of Isolates

In this study, we examined eight antibiotics that are highly important for both the clinical treatment of listeriosis and food safety concerns, including those related to the animal meat supply chain. Ampicillin and penicillin G are considered first-line options for invasive *L. monocytogenes* infections, often used in combination with an aminoglycoside such as gentamicin, and remain the mainstays of current treatment guidelines [1]. Trimethoprim–sulphamethoxazole (TMP-SMX) is recommended as an effective alternative for patients allergic to *β*-lactams and has been successfully used in documented cases of listeriosis [14,42]. Vancomycin is commonly used against multidrug-resistant (MDR) Gram-positive bacteria but is not a primary choice for listeriosis; including it helps us monitor susceptibility to a last-resort glycopeptide that is important in other important infections [43]. The remaining antibiotics—erythromycin, streptomycin, tetracycline, and chloramphenicol—represent additional drug classes that are used in specific clinical situations or have historically played a major role in human and veterinary medicine, including poultry production [44,45,46]. In particular, streptomycin was used as the representative aminoglycoside because it has been widely used in food animals and is frequently employed as a marker of aminoglycoside resistance in foodborne *L. monocytogenes* and other poultry-associated bacteria [47,48,49], while recognizing that gentamicin remains the preferred clinical aminoglycoside for combination therapy in invasive listeriosis. Thus, it should be noted that our panel was not intended to replicate standard treatment regimens but to cover both first-line anti-listerial agents and antibiotics that are historically or currently important in poultry production and in food-related AMR surveillance.

Disk diffusion testing revealed that most of the 93 *L. monocytogenes* isolates exhibited large inhibition zones against all eight antibiotics, with the majority falling into the 20–29 mm and 30–39 mm categories (87.3% overall across all drugs; Table 1). Only a very small percentage of isolates produced zones of 0–9 mm (1.7% across all antibiotics), and zones of 10–19 mm were also relatively rare except for streptomycin, where nearly two-thirds of isolates (62.4%) fell into this lower-range category. At the individual drug level, nearly all isolates showed zones of 20–39 mm for ampicillin, penicillin G, TMP-SMX, and tetracycline, and 20–29 mm for vancomycin, while distinct subpopulations with significantly smaller zones (0–9 mm) were observed for erythromycin (8.6%) and streptomycin (4.3%). Regarding chloramphenicol, a small percentage of isolates (7.5%) produced zones measuring 10–19 mm, while the remaining 92.5% fell within the 20–39 mm range. The heatmap in Table S2 reinforces this overall pattern of high susceptibility, with generally uniform, large inhibition zones for *β*-lactams (i.e., ampicillin and penicillin G), TMP-SMX, and tetracycline, and a more pronounced reduction in susceptibility among isolates for vancomycin, erythromycin, streptomycin, and chloramphenicol. Outliers presenting full resistance (no inhibition zone) were also evident for erythromycin and streptomycin.

**Figure 2:**
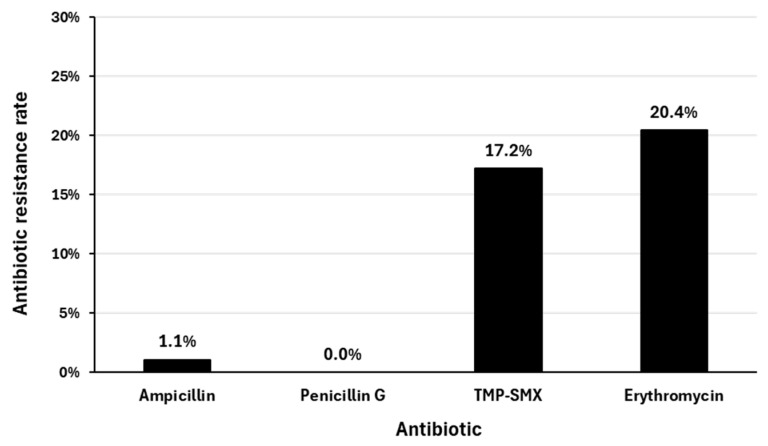
Antibiotic resistance rates of the *L. monocytogenes* isolates (n = 93) to ampicillin, penicillin G, TMP-SMX, and erythromycin, based on the EUCAST clinical breakpoint tables. No isolate exhibited resistance to penicillin G.

When inhibition zones were interpreted using EUCAST clinical breakpoints, resistance to first-line agents remained low, and no isolates were classified as resistant to penicillin G (Figure 2). However, a substantial proportion of isolates were classified as resistant to TMP-SMX and erythromycin (17.2% and 20.4%, respectively). These findings should, however, be interpreted with caution because the assay was performed under non-standardized conditions (with growth of the isolates on unsupplemented Mueller-Hinton agar after 24 h of incubation in ambient atmosphere). Future studies should confirm these findings using fully standardized EUCAST susceptibility testing conditions.

The persistent activity of ampicillin and penicillin G (benzylpenicillin) in our collection aligns with several large surveys of food and environmental *L. monocytogenes* across Europe, which generally report near-universal susceptibility to these primary drugs [50,51]. For instance, large-scale monitoring in France and Germany found that resistance to penicillin or ampicillin was observed only occasionally, and TMP-SMX also remained highly effective, with resistant strains accounting for at most a small percentage of all tested isolates [14,52]. Similar results have been seen for meat- and dairy-related isolates from Poland and Greece, with nearly all strains from meat products and soft cheeses, respectively, being susceptible to ampicillin, benzylpenicillin, and TMP-SMX [53,54]. Recent genome-based studies of large collections of *L. monocytogenes* also suggest that acquired resistance genes conferring *β*-lactam and TMP-SMX resistance remain rare, and that clinically significant resistance to these antibiotics remains exceptional in most industrialized environments [14,55,56]. In this regard, the absence of penicillin G resistance and the large zones of inhibition for ampicillin and TMP-SMX observed in most of our isolates indicate that, despite their food origin and potential drug exposure in the poultry farm environments, these strains have not yet experienced significant selection against the main antibiotics used to treat invasive listeriosis. However, the fact that 17.2% of isolates were resistant to TMP-SMX, according to EUCAST clinical breakpoint values, is concerning and underscores the need to remain vigilant.

For vancomycin, tetracycline, and chloramphenicol, inhibition zones generally also clustered into intermediate-to-large categories. Specifically, 95.7% of isolates showed 20–29 mm zones to vancomycin, and nearly all isolates displayed 20–39 mm zones to tetracycline and chloramphenicol (96.7% and 92.5%, respectively), with only small minorities in the 10–19 mm group (3.2% and 7.5%, respectively). This pattern aligns with long-term surveillance studies, which generally report full or near-full susceptibility of *L. monocytogenes* food isolates to vancomycin and low levels of resistance to tetracycline and chloramphenicol [12,54,57]. These latter resistant strains are usually a small subset carrying the *tet*(*M*) gene or chloramphenicol-resistance determinants (*cat*/*cmlA*) [19,58]. Although chloramphenicol is now banned in food-producing animals in Europe and many other countries worldwide, including poultry production, mainly due to its suspected carcinogenicity, residues of resistance genes persist in food-related environments [59]. Nevertheless, our data suggest that phenotypic resistance to this antibiotic is not common in the studied population. The symmetric distribution of tetracycline zones around 20–39 mm, with only 3.2% of isolates in the 10–19 mm range and none in the 0–9 mm range, also indicates limited current circulation of high-level tetracycline resistance among these poultry-derived isolates.

In contrast, erythromycin and streptomycin showed clearer signs of emerging resistant subpopulations, with 8.6% and 4.3% of isolates, respectively, displaying very small inhibition zones (0–9 mm), and a predominant 10–19 mm category for streptomycin (62.4%). Similar patterns have been observed in recent surveys of *L. monocytogenes* foodborne isolates, in which resistance to macrolides, lincosamides, and aminoglycosides was more common than resistance to *β*-lactams or TMP-SMX, often linked to mobile genetic elements carrying *erm*, *lnu*, or *str* genes [12,14,60,61]. For example, studies on RTE foods and vegetables have reported significant proportions of erythromycin- and streptomycin-resistant *L. monocytogenes* isolates, sometimes with MDR phenotypes involving macrolides, tetracyclines, and TMP-SMX [13,62]. The detection of a small subset of isolates with very small zones for erythromycin and streptomycin in our collection aligns with the broader pattern that resistance to macrolides and certain aminoglycosides is among the antibiotic classes most likely to develop and accumulate resistance in *L. monocytogenes*.

However, it is worth noting that the relatively favorable overall susceptibility profile observed here for ampicillin, penicillin, and TMP-SMX, contrasts with reports from several other regions of the world, where foodborne *L. monocytogenes* isolates show much higher resistance to these antibiotics as well [63,64]. Surveys of raw milk and other foods in Sri Lanka, Turkey, and parts of India, for example, have documented resistance levels of 40–80% for ampicillin and penicillin, along with significant proportions of strains resistant to macrolides (such as erythromycin) and folate pathway inhibitors (such as TMP-SMX), often with MDR indices exceeding 0.2 [64,65,66]. Meta-analyses and recent studies with large collections of foodborne *L. monocytogenes* isolates have further emphasized the emergence of an MDR phenotype in this pathogen across various food chains, with particularly high resistance to penicillin, TMP-SMX, and erythromycin in some settings [13,62,67,68]. These differences likely reflect variations in antibiotic use, enforcement of veterinary drug regulations, hygiene standards, and surveillance efforts across regions.

From the perspective of animal food systems, it is notable that the two antibiotics showing early signs of reduced susceptibility in our isolates—erythromycin and streptomycin—belong to classes (macrolides and aminoglycosides, respectively) that have been, or are still, widely used in poultry production and other animal husbandry [69,70,71]. Other studies on animal products, including poultry, have shown that these products can harbor *L. monocytogenes* and other foodborne pathogens with acquired resistance to tetracycline, TMP-SMX, and macrolides, suggesting that farm environments may serve as reservoirs and mixing grounds for resistance determinants [63,72,73]. This finding of reduced susceptibility to macrolides and aminoglycosides supports the idea that *L. monocytogenes* circulating in food chains may be exposed to selective pressures and may share resistance traits via mobile genetic elements. In this context, the generally high susceptibility to *β*-lactams and TMP-SMX is promising, but the erythromycin- and streptomycin-resistant phenotypes indicate that surveillance should remain vigilant.

### 2.3. No Correlation Between Biofilm-Forming Ability and Antibiotic Susceptibility

To explore potential links between surface-associated growth and antibiotic resistance, the biofilm-forming abilities of each isolate under the two tested conditions (BHI at 30 and 12 °C) were compared with the corresponding inhibition zone diameters for all eight antibiotics. A qualitative review of Tables S1 and S2 showed that isolates labeled as moderate biofilm producers in BHI at 12 °C (e.g., LFMH_B110–B114, B116, B121, B122, B126, B131, B142, B143, B186) generally showed inhibition zones similar to those of weak or non-biofilm formers across *β*-lactams, TMP-SMX, vancomycin, erythromycin, streptomycin, tetracycline, and chloramphenicol. Conversely, isolates with clearly smaller inhibition zones—such as those showing 0–9 mm for erythromycin or streptomycin, or an ampicillin zone of 0 mm (like LFMH_B118, B160, B165, B166, B171, B192, B198, B199)—were mainly classified as non- or weak biofilm formers, rather than as stronger biofilm producers. To support this qualitative assessment with formal statistical analysis, Spearman rank-order correlations were calculated between A_590 nm_ biofilm values and inhibition zone diameters for each antibiotic. Most correlations were weak and non-significant, indicating no consistent association between biofilm biomass and antibiotic susceptibility (Table S3). Only a few isolated weak positive correlations were observed, namely with penicillin G and biofilm formation at 30 °C (Spearman’s rho = 0.242, *p* = 0.019) and with chloramphenicol at both 30 °C (rho = 0.336, *p* = 0.001) and 12 °C (rho = 0.275, *p* = 0.008); however, these limited, small-magnitude associations were not reproduced across the other antibiotics and biofilm incubation conditions. Overall, these findings suggest that within this collection, increased biofilm biomass on PS does not consistently lead to smaller inhibition zones or to a more resistant phenotype against any of the antibiotics tested. Instead, susceptibility patterns appear largely independent of biofilm-forming capacity, with resistant or reduced-susceptibility profiles scattered among isolates spanning the full spectrum from non-biofilm to moderate biofilm formers.

From a broader food safety perspective, these findings reinforce the idea that biofilm formation and antibiotic resistance are related but separate aspects of *L. monocytogenes* persistence along animal-derived food chains. Indeed, previous studies have shown that isolates from food and processing environments can combine strong biofilm-forming ability with diverse virulence genes and traits, yet the strength of biofilm production does not always correlate with specific genomic patterns or lineages at the individual strain level [38,74,75]. At the same time, biofilms in farm environments are recognized as key hotspots for antimicrobial tolerance and for the horizontal transfer of resistance genes among coexisting bacteria, especially in multispecies biofilms, even when standard planktonic susceptibility tests still classify *L. monocytogenes* as susceptible [76,77]. Therefore, these findings should be considered when designing control strategies that address both antibiotic use and biofilm development along the food chain.

## 3. Materials and Methods

### 3.1. Bacterial Isolates and Preparation of Their Working Suspensions

The 93 *L. monocytogenes* isolates, along with their sources, are listed in Table S1. Most of them (n = 89; 95.7%) were isolated from raw chicken meat, while four were isolated from marinated chicken meat [20]. All isolates were derived from 36 different samples of chilled raw chicken meat, including whole carcasses, minced meat, and other parts of the broiler (such as breasts, thighs, wings, necks, and drumsticks). Meat samples were collected from three butcher shops and three supermarkets in Myrina, the main (capital) city of Lemnos Island, from July to October 2021. No serogroup or molecular-typing information is available for these isolates. All isolates were stored long-term at −80 °C in BHI broth (Lab M, Heywood, Lancashire, UK) containing 15% glycerol before analysis. To revive, a small volume (10–20 μL) of each was inoculated into 10 mL of Trypticasein Soy Broth (TSB; Condalab, Madrid, Spain) and incubated at 37 °C for 24 h. Then, 100 μL of this preculture was transferred to 10 mL of fresh TSB (1:100 dilution) and incubated at 37 °C for 18 h, producing a working culture of approximately 10^9^ CFU/mL. Both cultures were streaked onto Trypticasein Soy Agar (TSA; Condalab) to check for purity. A saline suspension of each isolate was prepared by centrifuging its working culture at 3000× *g* for 10 min. The supernatant was discarded, and the pellet was resuspended in ¼ Ringer’s solution (Lab M), then centrifuged again. After discarding the supernatant, 10 mL of ¼ Ringer’s were added to the pellet and stirred until dissolved. Finally, a 10 mL saline suspension for each isolate was adjusted to an absorbance of 0.1 at 600 nm (A_600 nm_), roughly 10^8^ CFU/mL, as verified by plate counting on TSA, and used as the inoculum in the subsequent biofilm experiments.

### 3.2. Biofilm Formation and Biomass Quantification

A protocol previously described by our team was used for biofilm formation and biomass quantification, with minor modifications [78]. Initially, 200 μL of each isolate’s saline suspension were transferred in duplicate to each well of a sterile 96-well PS microtiter plate (transparent, flat, Cat. No. 30096, SPL Life Sciences, Pocheon-si, Gyeonggi-do, Korea). The plate was incubated for 3 h at either 12 °C or 30 °C to promote initial cell attachment. These two temperatures were selected to represent chilled and severe-abuse conditions in food-processing environments. Immediately after the three-hour incubation, the bacterial suspensions were removed from the wells, and 200 μL of ¼ Ringer solution were added to each well to wash away loosely attached cells. The ¼ Ringer solution was then discarded, and 200 μL of BHI broth were added to each well. The plate was sealed with its lid, wrapped with paraffin tape to prevent evaporation, and incubated at either 12 °C or 30 °C for 120 and 48 h, respectively, to promote biofilm formation. The longer incubation at the lower temperature was necessary to allow sufficient bacterial growth. After the biofilm incubation period, the plates were taken out of the incubators, the planktonic cells were removed, and 200 μL of ¼ Ringer’s solution were added to each well to wash away loosely attached cells. This step was repeated after removing the solution with a 12-channel pipette. Next, 200 μL of 0.1% (*w*/*v*) crystal violet solution (Sigma-Aldrich Chemie GmbH, Taufkirchen, Germany) were added to each well, the plate was shaken, covered with aluminum foil, and incubated for 15 min at room temperature to stain the biofilm biomass. The crystal violet was then removed, discarded, and each well was rinsed thoroughly with deionized water to wash away excess dye. Immediately afterward, 200 μL of ethanol:acetone (80:20, *v*/*v*) were added (Sigma-Aldrich Chemie GmbH), the plates were shaken to solubilize the crystal violet bound to the biofilm, and incubated at 4 °C for 15 min. Finally, the absorbance (optical density) of the solution in each well was measured at 590 nm using a microplate reader (Tecan Spark^®^, Tecan Group Ltd., Männedorf, Switzerland) to indirectly estimate biofilm biomass. Wells filled with sterile ¼ Ringer’s solution underwent the same process as negative controls (NCs). Bacterial isolates were classified as non-biofilm formers or as weak, moderate, or strong biofilm formers using a previously described method [79]. Briefly, the cut-off optical density (ODc) was defined as the mean OD value of the NC plus three standard deviations (ODc = mean OD of NC + 3 × SD). Based on this threshold, isolates were classified as follows: OD ≤ ODc, non-biofilm former; ODc < OD ≤ 2 × ODc, weak biofilm former; 2 × ODc < OD ≤ 4 × ODc, moderate biofilm former; and OD > 4 × ODc, strong biofilm former. The mean ± SD values of the NCs under the two incubation conditions are provided in Table S1, together with the ODc values for each incubation condition (30 °C and 12 °C). Each experiment was conducted twice, starting with independent bacterial cultures.

### 3.3. Antibiotic Susceptibility Testing (Disk Diffusion Method)

Antibiotic susceptibility testing was performed using the disk diffusion method (Kirby-Bauer test). The panel included eight antibiotics selected to represent (a) first-line and clinically relevant anti-listerial agents (ampicillin, penicillin G, TMP-SMX, erythromycin), (b) antibiotic classes relevant to poultry production and surveillance (streptomycin as the aminoglycoside representative, tetracycline, chloramphenicol), and (c) a last-resort glycopeptide (vancomycin) to screen for potential emergence of glycopeptide non-susceptibility in food-chain isolates. To do the test, a 0.5 McFarland inoculum from each isolate working culture (10 mL in TSB) was spread on the surface of a dried (unsupplemented) Mueller-Hinton agar plate (Condalab) to achieve an initial population of about 10^7^ CFU. Six mm diameter paper discs (Thermo Scientific™ Oxoid™ Antimicrobial Susceptibility Test Discs, Oxoid Limited, Basingstoke, Hampshire, UK) impregnated with a specific amount of each of the following antibiotics: ampicillin (10 μg), penicillin G (benzylpenicillin) (10 μg), TMP-SMX (25 μg), vancomycin (30 μg), erythromycin (15 μg), streptomycin (10 μg), tetracycline (30 μg), and chloramphenicol (30 μg), were then placed onto the medium surface and incubated at 37 °C under ambient atmosphere for 24 h. At the end of incubation, each inhibition zone was measured twice on two plates, each inoculated with a different biological replicate of the target isolate working culture, at three different diameters per zone. The zone inhibition diameters (in mm) were categorized into six groups (1: 0–9 mm; 2: 10–19 mm; 3: 20–29 mm; 4: 30–39 mm; 5: 40–49 mm; and 6: 50–59 mm), and antibiotic effectiveness was finally expressed as the percentage of isolates in each category. Additionally, a heatmap was created using the Excel^®^ module of the Microsoft^®^ Office 365 suite (Redmond, WA, USA) to visualize the results across all isolates and antibiotics. Results for ampicillin, penicillin G, TMP-SMX, and erythromycin were also interpreted based on the EUCAST Clinical Breakpoint Tables v. 16.0 (valid from 1 January 2026), categorizing isolates as resistant (R) or sensitive (S), since clinical breakpoint values are available for these against *L. monocytogenes*. However, because the disk diffusion assay was performed under non-standardized conditions, the zone diameter results are presented as screening data and should not be interpreted as fully EUCAST-compliant categorical susceptibility results.

### 3.4. Statistics

Spearman’s rank-order correlation analysis was used to assess the relationship between biofilm biomass values (A_590 nm_) obtained for each isolate under two incubation conditions: 30 °C for 48 h and 12 °C for 120 h. The same analysis was further applied to evaluate the association between biofilm biomass values and the corresponding mean inhibition zone diameters (mm) for each of the eight tested antibiotics. Spearman’s rho (*ρ*) was selected as a non-parametric measure of association appropriate for assessing monotonic relationships between variables without assuming normality. All tests were two-tailed and performed using IBM SPSS Statistics (v.23; IBM Corporation, Armonk, NY, USA), and statistical significance was set at *p* < 0.05.

## 4. Conclusions and Future Perspectives

The present work shows that most *L. monocytogenes* poultry isolates tested here display only weak-to-moderate biofilm-forming capacity on PS, and that this phenotype is influenced by incubation temperature and time (at 30 °C for 48 h; at 12 °C for 120 h). Nevertheless, even these relatively weak biofilms, particularly when combined with complex surface topographies, conditioning films, and resident microbiota in real-world processing environments, may still contribute to persistence during cleaning and disinfection in meat supply chains. At the same time, the overall susceptibility of the isolates to first-line anti-listerial antibiotics (ampicillin, penicillin G, and TMP-SMX) and to vancomycin, tetracycline, and chloramphenicol is reassuring. However, the detection of erythromycin- and streptomycin-resistant or low-susceptibility subpopulations, and the lack of a consistent link between biofilm-forming ability and antibiotic resistance, highlight the need for ongoing surveillance and suggest that biofilm formation and antibiotic resistance are likely related but mostly independent aspects of *L. monocytogenes* persistence. In practical terms, these findings underscore the importance of integrated control approaches that combine stringent hygiene and sanitation programs targeted at biofilms (including validation of disinfectants against sessile cells), equipment and surface designs that minimize niches for biofilm development, and systematic environmental monitoring for *L. monocytogenes* and resistance phenotypes in processing facilities. Prudent antimicrobial use along the poultry production chain is also essential to reduce selection pressure for resistant strains that may subsequently colonize food-contact surfaces.

In summary, these results support the use of integrated *Listeria* control plans in poultry processing. Undoubtedly, our findings should be interpreted as specific to the tested poultry-derived isolates and the in vitro monoculture conditions used here; they may not fully reflect behavior in complex, real-world processing environments, where broader patterns of persistence and susceptibility may differ due to multispecies interactions, surface properties, and other environmental factors. Future studies could expand this phenotypic characterization to include in situ plant isolates, multispecies biofilms, and genomic factors associated primarily with macrolide and aminoglycoside resistance, thereby better informing risk assessment and control strategies in poultry processing environments. In addition, future work should evaluate antibiotic susceptibility in representative isolates under true biofilm conditions to better assess the clinical and food-safety relevance of these phenotypes, as biofilm-associated cells can exhibit distinct susceptibility profiles.

## Figures and Tables

**Table 1:** Percentage of *L. monocytogenes* isolates (n = 93) across six antibiotic susceptibility categories, based on zone inhibition diameters (1: 0–9 mm; 2: 10–19 mm; 3: 20–29 mm; 4: 30–39 mm; 5: 40–49 mm; and 6: 50–59 mm), for each of the eight tested antibiotics.

Inhibition Zone Diameter (mm)	Ampicillin	Penicillin G	TMP-SMX	Vancomycin	Erythromycin	Streptomycin	Tetracycline	Chloramphenicol	All Tested Antibiotics
0–9	1.1%	0.0%	0.0%	0.0%	8.6%	4.3%	0.0%	0.0%	1.7%
10–19	0.0%	0.0%	0.0%	4.3%	1.1%	62.4%	3.2%	7.5%	9.8%
20–29	11.8%	25.8%	19.4%	95.7%	30.1%	33.3%	46.2%	51.6%	39.2%
30–39	81.7%	72.0%	79.6%	0.0%	60.2%	0.0%	50.5%	40.9%	48.1%
40–49	4.3%	2.2%	1.1%	0.0%	0.0%	0.0%	0.0%	0.0%	0.9%
50–59	1.1%	0.0%	0.0%	0.0%	0.0%	0.0%	0.0%	0.0%	0.1%

## Data Availability

The data presented in this study are contained within the article.

## Supplementary Materials

The following supporting information can be downloaded at: https://www.mdpi.com/article/10.3390/antibiotics15060577/s1, Table S1: Biofilm biomasses (A590 nm) of the 93 L. monocytogenes isolates and classification of each isolate as non-biofilm former, weak, or moderate biofilm former. Each isolate was allowed to form biofilm on the surface of the PS microtiter plate during incubation in BHI broth at 30 or 12 °C for 48 or 120 h, respectively. The biofilm absorbance data are expressed as mean ± standard deviation. Values for the negative controls (NCs) are also included, along with the cut-off optical density (ODc) values for each incubation condition; Table S2: Heatmap showing the susceptibilities of the 93 L. monocytogenes isolates to the eight tested antibiotics, based on mean inhibition zone diameters (in mm). The legend explaining the color range is located below the table; Table S3: Results of Spearman’s rank-order correlation analyses assessing the association: (i) between biofilm biomass values (A590 nm) obtained for each isolate under two incubation conditions: 30 °C for 48 h and 12 °C for 120 h, and (ii) between biofilm biomass values and the corresponding mean inhibition zone diameters (mm) for each of the eight tested antibiotics.

## Abbreviations

The following abbreviations are used in this manuscript:

AMR	Antimicrobial resistance
BHI	Brain Heart Infusion
CFU	Colony-Forming Unit
EUCAST	European Committee on Antimicrobial Susceptibility Testing
LFMH	Laboratory of Food Microbiology and Hygiene
MDR	Multidrug-resistant or Multidrug resistance
PS	Polystyrene
RTE	Ready to eat
TMP-SMX	Trimethoprim–sulphamethoxazole
TSA	Trypticasein Soy Agar
TSB	Trypticasein Soy Broth
