# Supplementary material for: Phenotypic Profiling of Biofilm Formation and Antibiotic Susceptibility in Poultry-Derived Listeria monocytogenes Isolates
Source: Antibiotics (Basel). 2026 Jun 5;15(6):577. doi: 10.3390/antibiotics15060577 (PMC13295242; doi:10.3390/antibiotics15060577)
Supplement: Supplementary file 1 [file antibiotics-15-00577-s001.zip › Table S1.pdf]

Table S1. Biofilm biomasses ($A_{590\ nm}$) of the 93 *L. monocytogenes* isolates and classification of each isolate as non-biofilm former, weak, or moderate biofilm former. Each isolate was allowed to form biofilm on the surface of the PS microtiter plate during incubation in BHI broth at 30 or 12 °C for 48 or 120 h, respectively. The biofilm absorbance data are expressed as mean ± standard deviation. Values for the negative controls (NCs) are also included, along with the cut-off optical density (ODc) values for each incubation condition.

s/n	Isolate code	Isolation origin	Meat sample code	30 °C (48 h)		12 °C (120 h)	
				$A_{590\ nm}$	Characterization	$A_{590\ nm}$	Characterization
1	**LFMH_B107**	raw chicken meat	1	0.54 ± 0.13	weak biofilm former	0.49 ± 0.16	weak biofilm former
2	**LFMH_B108**	raw chicken meat	1	0.69 ± 0.46	weak biofilm former	0.58 ± 0.27	weak biofilm former
3	**LFMH_B109**	raw chicken meat	1	0.63 ± 0.28	weak biofilm former	0.55 ± 0.21	weak biofilm former
4	**LFMH_B110**	raw chicken meat	1	0.61 ± 0.24	weak biofilm former	0.62 ± 0.29	moderate biofilm former
5	**LFMH_B111**	raw chicken meat	6	0.63 ± 0.25	weak biofilm former	0.65 ± 0.16	moderate biofilm former
6	**LFMH_B112**	raw chicken meat	6	0.65 ± 0.22	weak biofilm former	0.68 ± 0.15	moderate biofilm former
7	**LFMH_B113**	raw chicken meat	6	0.68 ± 0.21	weak biofilm former	0.66 ± 0.28	moderate biofilm former
8	**LFMH_B114**	raw chicken meat	6	0.65 ± 0.17	weak biofilm former	0.76 ± 0.37	moderate biofilm former
9	**LFMH_B115**	raw chicken meat	12	0.51 ± 0.12	weak biofilm former	0.43 ± 0.15	weak biofilm former
10	**LFMH_B116**	raw chicken meat	12	0.52 ± 0.10	weak biofilm former	0.65 ± 0.27	moderate biofilm former
11	**LFMH_B117**	raw chicken meat	12	0.56 ± 0.14	weak biofilm former	0.58 ± 0.19	weak biofilm former
12	**LFMH_B118**	raw chicken meat	14	0.60 ± 0.14	weak biofilm former	0.44 ± 0.09	weak biofilm former
13	**LFMH_B119**	raw chicken meat	12	0.51 ± 0.12	weak biofilm former	0.56 ± 0.23	weak biofilm former
14	**LFMH_B120**	raw chicken meat	12	0.51 ± 0.08	weak biofilm former	0.55 ± 0.28	weak biofilm former
15	**LFMH_B121**	raw chicken meat	12	0.54 ± 0.11	weak biofilm former	0.61 ± 0.26	moderate biofilm former
16	**LFMH_B122**	raw chicken meat	17	0.44 ± 0.12	weak biofilm former	0.63 ± 0.33	moderate biofilm former
17	**LFMH_B123**	raw chicken meat	17	0.45 ± 0.11	weak biofilm former	0.48 ± 0.14	weak biofilm former
18	**LFMH_B124**	raw chicken meat	16	0.56 ± 0.14	weak biofilm former	0.54 ± 0.18	weak biofilm former
19	**LFMH_B125**	raw chicken meat	17	0.47 ± 0.12	weak biofilm former	0.56 ± 0.20	weak biofilm former
20	**LFMH_B126**	raw chicken meat	16	0.56 ± 0.14	weak biofilm former	0.61 ± 0.27	moderate biofilm former

21	**LFMH_B127**	raw chicken meat	16	0.57 ± 0.12	weak biofilm former	0.52 ± 0.22	weak biofilm former
22	**LFMH_B128**	raw chicken meat	17	0.41 ± 0.09	weak biofilm former	0.52 ± 0.23	weak biofilm former
23	**LFMH_B129**	raw chicken meat	17	0.46 ± 0.11	weak biofilm former	0.51 ± 0.19	weak biofilm former
24	**LFMH_B130**	raw chicken meat	16	0.57 ± 0.17	weak biofilm former	0.55 ± 0.18	weak biofilm former
25	**LFMH_B131**	raw chicken meat	21	0.68 ± 0.13	weak biofilm former	0.60 ± 0.13	moderate biofilm former
26	**LFMH_B132**	raw chicken meat	23	0.26 ± 0.06	non-biofilm former	0.23 ± 0.04	non-biofilm former
27	**LFMH_B133**	raw chicken meat	23	0.54 ± 0.20	weak biofilm former	0.43 ± 0.18	weak biofilm former
28	**LFMH_B134**	raw chicken meat	21	0.60 ± 0.13	weak biofilm former	0.63 ± 0.23	moderate biofilm former
29	**LFMH_B135**	raw chicken meat	21	0.60 ± 0.12	weak biofilm former	0.61 ± 0.26	moderate biofilm former
30	**LFMH_B136**	raw chicken meat	22	0.49 ± 0.09	weak biofilm former	0.44 ± 0.23	weak biofilm former
31	**LFMH_B137**	raw chicken meat	22	0.72 ± 0.45	weak biofilm former	0.40 ± 0.11	weak biofilm former
32	**LFMH_B138**	raw chicken meat	23	0.34 ± 0.19	non-biofilm former	0.22 ± 0.03	non-biofilm former
33	**LFMH_B139**	raw chicken meat	23	0.29 ± 0.13	non-biofilm former	0.21 ± 0.03	non-biofilm former
34	**LFMH_B140**	raw chicken meat	21	0.58 ± 0.14	weak biofilm former	0.54 ± 0.07	weak biofilm former
35	**LFMH_B141**	raw chicken meat	22	0.43 ± 0.11	weak biofilm former	0.31 ± 0.07	weak biofilm former
36	**LFMH_B142**	raw chicken meat	24	0.75 ± 0.14	weak biofilm former	0.65 ± 0.11	moderate biofilm former
37	**LFMH_B143**	raw chicken meat	27	0.69 ± 0.16	weak biofilm former	0.65 ± 0.13	moderate biofilm former
38	**LFMH_B144**	raw chicken meat	27	0.64 ± 0.20	weak biofilm former	0.56 ± 0.14	weak biofilm former
39	**LFMH_B145**	raw chicken meat	29	0.44 ± 0.08	weak biofilm former	0.44 ± 0.10	weak biofilm former
40	**LFMH_B146**	raw chicken meat	29	0.63 ± 0.13	weak biofilm former	0.53 ± 0.09	weak biofilm former
41	**LFMH_B147**	raw chicken meat	32	0.44 ± 0.10	weak biofilm former	0.46 ± 0.10	weak biofilm former
42	**LFMH_B148**	raw chicken meat	32	0.44 ± 0.20	weak biofilm former	0.49 ± 0.11	weak biofilm former
43	**LFMH_B149**	raw chicken meat	36	0.48 ± 0.11	weak biofilm former	0.43 ± 0.13	weak biofilm former
44	**LFMH_B150**	raw chicken meat	32	0.48 ± 0.10	weak biofilm former	0.48 ± 0.09	weak biofilm former
45	**LFMH_B151**	raw chicken meat	37	0.31 ± 0.05	non-biofilm former	0.25 ± 0.04	non-biofilm former
46	**LFMH_B152**	raw chicken meat	37	0.35 ± 0.10	non-biofilm former	0.27 ± 0.06	non-biofilm former
47	**LFMH_B153**	raw chicken meat	37	0.49 ± 0.13	weak biofilm former	0.40 ± 0.13	weak biofilm former
48	**LFMH_B154**	raw chicken meat	37	0.53 ± 0.20	weak biofilm former	0.43 ± 0.14	weak biofilm former

49	**LFMH_B155**	raw chicken meat	37	0.46 ± 0.08	weak biofilm former	0.45 ± 0.09	weak biofilm former
50	**LFMH_B156**	raw chicken meat	37	0.36 ± 0.14	non-biofilm former	0.26 ± 0.04	non-biofilm former
51	**LFMH_B157**	raw chicken meat	37	0.41 ± 0.04	weak biofilm former	0.46 ± 0.08	weak biofilm former
52	**LFMH_B158**	raw chicken meat	44	0.28 ± 0.11	non-biofilm former	0.22 ± 0.05	non-biofilm former
53	**LFMH_B159**	raw chicken meat	44	0.24 ± 0.09	non-biofilm former	0.20 ± 0.04	non-biofilm former
54	**LFMH_B160**	raw chicken meat	45	0.27 ± 0.08	non-biofilm former	0.19 ± 0.04	non-biofilm former
55	**LFMH_B161**	raw chicken meat	45	0.31 ± 0.15	non-biofilm former	0.19 ± 0.04	non-biofilm former
56	**LFMH_B162**	raw chicken meat	46	0.58 ± 0.11	weak biofilm former	0.59 ± 0.17	weak biofilm former
57	**LFMH_B163**	raw chicken meat	46	0.41 ± 0.13	weak biofilm former	0.58 ± 0.20	weak biofilm former
58	**LFMH_B164**	raw chicken meat	44	0.46 ± 0.51	weak biofilm former	0.18 ± 0.03	non-biofilm former
59	**LFMH_B165**	raw chicken meat	46	0.53 ± 0.12	weak biofilm former	0.53 ± 0.10	weak biofilm former
60	**LFMH_B166**	raw chicken meat	46	0.28 ± 0.11	non-biofilm former	0.28 ± 0.07	weak biofilm former
61	**LFMH_B167**	raw chicken meat	47	0.50 ± 0.09	weak biofilm former	0.52 ± 0.11	weak biofilm former
62	**LFMH_B168**	raw chicken meat	48	0.60 ± 0.20	weak biofilm former	0.51 ± 0.10	weak biofilm former
63	**LFMH_B169**	raw chicken meat	47	0.70 ± 0.32	weak biofilm former	0.53 ± 0.12	weak biofilm former
64	**LFMH_B170**	raw chicken meat	48	0.56 ± 0.11	weak biofilm former	0.48 ± 0.10	weak biofilm former
65	**LFMH_B171**	raw chicken meat	48	0.53 ± 0.15	weak biofilm former	0.43 ± 0.11	weak biofilm former
66	**LFMH_B172**	raw chicken meat	49	0.39 ± 0.04	non-biofilm former	0.43 ± 0.12	weak biofilm former
67	**LFMH_B173**	raw chicken meat	49	0.38 ± 0.03	non-biofilm former	0.47 ± 0.09	weak biofilm former
68	**LFMH_B174**	raw chicken meat	52	0.44 ± 0.08	weak biofilm former	0.39 ± 0.05	weak biofilm former
69	**LFMH_B175**	raw chicken meat	52	0.53 ± 0.13	weak biofilm former	0.45 ± 0.16	weak biofilm former
70	**LFMH_B176**	raw chicken meat	52	0.48 ± 0.06	weak biofilm former	0.39 ± 0.03	weak biofilm former
71	**LFMH_B177**	raw chicken meat	55	0.28 ± 0.07	non-biofilm former	0.29 ± 0.08	non-biofilm former
72	**LFMH_B178**	raw chicken meat	55	0.34 ± 0.04	non-biofilm former	0.28 ± 0.08	non-biofilm former
73	**LFMH_B179**	raw chicken meat	60	0.41 ± 0.10	weak biofilm former	0.44 ± 0.13	weak biofilm former
74	**LFMH_B180**	raw chicken meat	57	0.59 ± 0.25	weak biofilm former	0.48 ± 0.08	weak biofilm former
75	**LFMH_B181**	raw chicken meat	60	0.47 ± 0.15	weak biofilm former	0.52 ± 0.08	weak biofilm former
76	**LFMH_B182**	raw chicken meat	61	0.59 ± 0.16	weak biofilm former	0.50 ± 0.06	weak biofilm former

77	**LFMH_B183**	raw chicken meat	61	0.56 ± 0.11	weak biofilm former	0.47 ± 0.07	weak biofilm former
78	**LFMH_B184**	raw chicken meat	64	0.44 ± 0.06	weak biofilm former	0.52 ± 0.12	weak biofilm former
79	**LFMH_B185**	raw chicken meat	64	0.55 ± 0.05	weak biofilm former	0.53 ± 0.12	weak biofilm former
80	**LFMH_B186**	marinated chicken	20	0.56 ± 0.18	weak biofilm former	0.66 ± 0.13	moderate biofilm former
81	**LFMH_B191**	raw chicken meat	18	0.28 ± 0.04	non-biofilm former	0.25 ± 0.07	non-biofilm former
82	**LFMH_B192**	raw chicken meat	35	0.30 ± 0.14	non-biofilm former	0.24 ± 0.06	non-biofilm former
83	**LFMH_B193**	raw chicken meat	41	0.30 ± 0.07	non-biofilm former	0.24 ± 0.08	non-biofilm former
84	**LFMH_B194**	raw chicken meat	37	0.29 ± 0.05	non-biofilm former	0.27 ± 0.09	non-biofilm former
85	**LFMH_B195**	raw chicken meat	38	0.24 ± 0.01	non-biofilm former	0.27 ± 0.11	non-biofilm former
86	**LFMH_B196**	raw chicken meat	42	0.29 ± 0.05	non-biofilm former	0.26 ± 0.04	non-biofilm former
87	**LFMH_B197**	raw chicken meat	43	0.26 ± 0.06	non-biofilm former	0.18 ± 0.02	non-biofilm former
88	**LFMH_B198**	raw chicken meat	53	0.28 ± 0.04	non-biofilm former	0.22 ± 0.04	non-biofilm former
89	**LFMH_B199**	raw chicken meat	53	0.31 ± 0.07	non-biofilm former	0.22 ± 0.05	non-biofilm former
90	**LFMH_B200**	marinated chicken	20	0.26 ± 0.05	non-biofilm former	0.24 ± 0.08	non-biofilm former
91	**LFMH_B201**	marinated chicken	54	0.20 ± 0.03	non-biofilm former	0.28 ± 0.09	non-biofilm former
92	**LFMH_B202**	marinated chicken	54	0.21 ± 0.05	non-biofilm former	0.22 ± 0.02	non-biofilm former
93	**LFMH_B203**	raw chicken meat	55	0.22 ± 0.05	non-biofilm former	0.21 ± 0.04	non-biofilm former
	**NC**			0.20 ± 0.06		0.17 ± 0.04	

**ODc (30 °C)**	**0.39**		
non-biofilm former	0.39	≥OD	
weak biofilm former	0.39	<OD≤	0.78
moderate biofilm former	0.78	<OD≤	1.56
strong biofilm former	1.56	<OD	

**ODc (12 °C)**	**0.29**		
non-biofilm former	0.29	≥OD	
weak biofilm former	0.29	<OD≤	0.59
moderate biofilm former	0.59	<OD≤	1.18
strong biofilm former	1.18	<OD	
