# Supplementary material for: Phenotypic Profiling of Biofilm Formation and Antibiotic Susceptibility in Poultry-Derived Listeria monocytogenes Isolates
Source: Antibiotics (Basel). 2026 Jun 5;15(6):577. doi: 10.3390/antibiotics15060577 (PMC13295242; doi:10.3390/antibiotics15060577)
Supplement: Supplementary file 1 [file antibiotics-15-00577-s001.zip › Table S2.pdf]

Table S2. Heatmap showing the susceptibilities of the 93 *L. monocytogenes* isolates to the eight tested antibiotics, based on mean inhibition zone diameters (in mm). The legend explaining the color range is located below the table.

Isolate	Ampicillin	Penicillin G	TMP-SMX	Vancomycin	Erythromycin	Streptomycin	Tetracycline	Chloramphenicol
LFMH_B107	37	39	36	24	36	21	34	33
LFMH_B108	38	37	37	24	35	19	36	35
LFMH_B109	37	37	36	24	33	22	33	31
LFMH_B110	35	35	32	23	31	18	31	32
LFMH_B111	33	31	31	22	29	18	27	27
LFMH_B112	30	29	32	20	26	17	28	23
LFMH_B113	37	32	34	25	34	20	32	31
LFMH_B114	38	33	34	24	32	19	32	32
LFMH_B115	34	32	32	23	29	17	26	28
LFMH_B116	34	34	32	22	31	18	27	30
LFMH_B117	34	34	34	22	30	14	30	30
LFMH_B118	0	38	36	25	35	20	34	33
LFMH_B119	35	30	33	21	30	18	24	27
LFMH_B120	29	29	28	20	24	14	24	22
LFMH_B121	31	32	33	22	33	18	31	27
LFMH_B122	35	34	34	23	31	18	32	31
LFMH_B123	32	32	32	21	29	17	28	28
LFMH_B124	39	38	38	26	35	20	34	34
LFMH_B125	33	32	32	21	28	17	28	28
LFMH_B126	37	35	34	24	34	19	32	30
LFMH_B127	38	36	38	25	34	19	33	33
LFMH_B128	36	36	34	24	32	18	30	32
LFMH_B129	40	38	38	26	37	23	36	34
LFMH_B130	34	30	30	21	25	15	24	26
LFMH_B131	36	36	36	24	33	18	32	32
LFMH_B132	38	35	34	24	33	21	32	29
LFMH_B133	38	38	37	24	36	21	33	33
LFMH_B134	37	39	37	26	37	20	35	32
LFMH_B135	38	39	38	26	37	20	36	34
LFMH_B136	34	34	31	21	31	16	30	30
LFMH_B137	34	35	30	22	31	19	30	31
LFMH_B138	36	35	34	23	34	21	31	26
LFMH_B139	34	30	32	22	31	17	28	26
LFMH_B140	37	32	36	26	35	20	32	32
LFMH_B141	37	34	36	24	33	20	30	31
LFMH_B142	33	32	33	26	31	20	28	31

LFMH_B143	39	38	36	27	35	20	33	34
LFMH_B144	39	38	35	23	36	20	34	34
LFMH_B145	36	31	34	23	31	17	30	29
LFMH_B146	32	31	31	21	29	17	28	27
LFMH_B147	34	35	32	25	33	19	32	32
LFMH_B148	36	38	35	26	36	21	32	31
LFMH_B149	36	35	36	25	35	20	33	30
LFMH_B150	35	28	31	20	26	17	25	26
LFMH_B151	37	35	36	26	35	20	30	30
LFMH_B152	40	37	38	26	37	23	33	29
LFMH_B153	38	36	36	24	33	20	31	31
LFMH_B154	39	37	36	25	35	21	33	31
LFMH_B155	40	40	37	26	38	22	35	36
LFMH_B156	27	27	29	21	25	14	23	21
LFMH_B157	31	30	32	20	27	18	26	27
LFMH_B158	30	26	28	20	25	16	25	22
LFMH_B159	30	31	28	20	24	16	27	24
LFMH_B160	55	24	25	19	0	13	22	17
LFMH_B161	26	25	25	20	20	12	26	15
LFMH_B162	28	27	30	22	24	14	22	24
LFMH_B163	27	28	26	18	22	13	21	19
LFMH_B164	29	29	25	20	25	14	22	22
LFMH_B165	29	28	27	20	0	12	24	17
LFMH_B166	29	26	25	20	0	12	27	15
LFMH_B167	30	25	27	20	19	11	23	22
LFMH_B168	30	29	28	19	0	13	20	20
LFMH_B169	33	33	31	23	26	14	26	31
LFMH_B170	30	30	30	19	0	12	24	20
LFMH_B171	32	30	30	20	0	13	23	26
LFMH_B172	31	29	32	20	23	11	25	29
LFMH_B173	30	28	30	21	28	0	23	29
LFMH_B174	30	27	30	20	0	13	25	27
LFMH_B175	29	27	30	20	20	12	24	26
LFMH_B176	33	30	29	21	25	15	25	28
LFMH_B177	36	35	35	25	33	21	32	27
LFMH_B178	37	36	35	25	35	21	33	29
LFMH_B179	38	37	36	23	34	21	33	29
LFMH_B180	38	37	35	23	35	21	32	31
LFMH_B181	37	34	35	29	34	19	31	30
LFMH_B182	26	23	25	20	20	12	20	22
LFMH_B183	30	31	32	21	30	13	27	23
LFMH_B184	34	30	31	20	27	12	25	18
LFMH_B185	32	30	30	21	24	13	25	20
LFMH_B186	30	27	26	20	0	12	23	19
LFMH_B191	25	25	28	20	26	15	24	23
LFMH_B192	34	31	31	26	31	0	14	27

LFMH_B193	31	29	28	22	27	15	26	26
LFMH_B194	31	23	33	24	31	21	30	27
LFMH_B195	36	28	30	23	25	13	25	26
LFMH_B196	41	40	41	28	38	23	36	33
LFMH_B197	31	31	29	22	30	18	29	26
LFMH_B198	36	34	35	23	33	0	15	28
LFMH_B199	35	33	34	25	33	0	14	29
LFMH_B200	32	30	33	24	31	18	30	27
LFMH_B201	33	31	32	24	32	19	30	27
LFMH_B202	36	34	36	24	34	21	33	26
LFMH_B203	36	35	35	25	34	21	34	33

Inhibition zone diameter (mm)
0 - 9
10 - 19
20 - 29
30 - 39
40 -49
50 - 59
