# Supplementary material for: Phenotypic Profiling of Biofilm Formation and Antibiotic Susceptibility in Poultry-Derived Listeria monocytogenes Isolates
Source: Antibiotics (Basel). 2026 Jun 5;15(6):577. doi: 10.3390/antibiotics15060577 (PMC13295242; doi:10.3390/antibiotics15060577)
Supplement: Supplementary file 1 [file antibiotics-15-00577-s001.zip › Table S3.pdf]

Table S3. Results of Spearman's rank-order correlation analyses assessing the association: (i) between biofilm biomass values ($A_{590\ nm}$) obtained for each isolate under two incubation conditions: 30 °C for 48 h and 12 °C for 120 h, and (ii) between biofilm biomass values and the corresponding mean inhibition zone diameters (mm) for each of the eight tested antibiotics.

Correlations

			Biofilm (30 °C)	Biofilm (12 °C)	Ampicillin	PenicillinG	TMP-SMX	Vancomycin	Erythromycin	Streptomycin	Tetracycline	Chloramphenicol
Spearman's rho	Biofilm (30 °C)	Correlation Coefficient	1.000	0.794**	0.078	0.242*	0.153	0.055	0.091	0.066	0.168	0.336**
		Sig. (2-tailed)	0.000	0.000	0.454	0.019	0.142	0.603	0.385	0.528	0.108	0.001
		N	93	93	93	93	93	93	93	93	93	93
	Biofilm (12 °C)	Correlation Coefficient	0.794**	1.000	0.074	0.168	0.147	0.048	0.061	0.041	0.153	0.275**
		Sig. (2-tailed)	0.000	0.000	0.479	0.107	0.161	0.650	0.562	0.699	0.143	0.008
		N	93	93	93	93	93	93	93	93	93	93
	Ampicillin	Correlation Coefficient	0.078	0.074	1.000	0.771**	0.798**	0.712**	0.784**	0.697**	0.704**	0.711**
		Sig. (2-tailed)	0.454	0.479	0.000	0.000	0.000	0.000	0.000	0.000	0.000	0.000
		N	93	93	93	93	93	93	93	93	93	93
	PenicillinG	Correlation Coefficient	0.242*	0.168	0.771**	1.000	0.873**	0.778**	0.897**	0.770**	0.848**	0.854**
		Sig. (2-tailed)	0.019	0.107	0.000	0.000	0.000	0.000	0.000	0.000	0.000	0.000
		N	93	93	93	93	93	93	93	93	93	93
	TMP-SMX	Correlation Coefficient	0.153	0.147	0.798**	0.873**	1.000	0.836**	0.921**	0.791**	0.842**	0.821**
		Sig. (2-tailed)	0.142	0.161	0.000	0.000	0.000	0.000	0.000	0.000	0.000	0.000
		N	93	93	93	93	93	93	93	93	93	93
	Vancomycin	Correlation Coefficient	0.055	0.048	0.712**	0.778**	0.836**	1.000	0.884**	0.735**	0.741**	0.782**

	Sig. (2-tailed)	0.603	0.650	0.000	0.000	0.000	0.000	0.000	0.000	0.000	0.000
	N	93	93	93	93	93	93	93	93	93	93
Erythromycin	Correlation Coefficient	0.091	0.061	0.784**	0.897**	0.921**	0.884**	1.000	0.841**	0.864**	0.818**
	Sig. (2-tailed)	0.385	0.562	0.000	0.000	0.000	0.000	0.000	0.000	0.000	0.000
	N	93	93	93	93	93	93	93	93	93	93
Streptomycin	Correlation Coefficient	0.066	0.041	0.697**	0.770**	0.791**	0.735**	0.841**	1.000	0.879**	0.680**
	Sig. (2-tailed)	0.528	0.699	0.000	0.000	0.000	0.000	0.000	0.000	0.000	0.000
	N	93	93	93	93	93	93	93	93	93	93
Tetracycline	Correlation Coefficient	0.168	0.153	0.704**	0.848**	0.842**	0.741**	0.864**	0.879**	1.000	0.787**
	Sig. (2-tailed)	0.108	0.143	0.000	0.000	0.000	0.000	0.000	0.000	0.000	0.000
	N	93	93	93	93	93	93	93	93	93	93
Chloramphenicol	Correlation Coefficient	0.336**	0.275**	0.711**	0.854**	0.821**	0.782**	0.818**	0.680**	0.787**	1.000
	Sig. (2-tailed)	0.001	0.008	0.000	0.000	0.000	0.000	0.000	0.000	0.000	0.000
	N	93	93	93	93	93	93	93	93	93	93

**. Correlation is significant at the 0.01 level (2-tailed).

*. Correlation is significant at the 0.05 level (2-tailed).
